# SymptomSpeak: Women’s Struggle for History and Health in Kosovo

**DOI:** 10.1007/s11013-021-09746-1

**Published:** 2021-08-31

**Authors:** Hanna Kienzler

**Affiliations:** Department of Global Health & Social Medicine, Faculty of Social Science and Public Policy, School of Global Affairs, King’s College London, 40 Aldwych; Bush House (NE) 3.15, London, WC2B 4BG UK

**Keywords:** Mental health, Symptoms, Pain, War, Kosovo

## Abstract

What are the linguistic dimensions of pain, and what kind of articulations arise from these painful experiences? How does the language of pain circulate, connect, and reach across histories, gendered realities, and social politics? In what ways might the language of pain act on and transform the world by shaping and changing socio-political agendas? I explored these questions among women in Kosovo and discovered a unique symptomatic language which I call SymptomSpeak. SymptomSpeak is a powerful language evoked, shared, and exchanged by women to articulate political, social, and economic grievances, to challenge societal norms, and to demand justice. The language itself consists of a detailed symptom vocabulary which is variously assembled into meaning complexes. Such assemblages shift depending on the social context in which they are conveyed and are referred to as *nervoz* (nervousness), *mërzitna* (worried, sad), *mzysh* (evil eye), and *t’bone* (spell). I describe in detail how women variously combine and exchange components of SymptomSpeak and, thereby, question dominant framings of reality. Thereby, my intention is to contribute to a new understanding of pain as language which straddles the fine line between socio-political commentary and illness; produces gendered political realities; and challenges the status quo through its communicative power.

## Introduction

Elona^1^ interrupted our conversation touching her neck and moaning “ah, what a headache…” For two days, she had suffered from strong pains “from the top of the head down to the neck.” That day, she had visited the village doctor who had checked her heart and pulse but could not find anything abnormal and recommended aspirin. Elona knew well where her frequently experienced headaches had originated and reflected on this when she told me, “When the [mortal] remains of my husband were returned to us two years ago, my daughter had great problems. As she was hearing the news, she screamed, threw her arms into the air, she couldn’t control herself anymore.” Upon seeing her daughter’s suffering, it was as if her pain had relocated itself into Elona’s body, expressing itself through great emotional distress and physical symptoms. “I became so worried and nervous that my heart started racing, my blood [blood pressure] went up, and I got this enormous headache,” she said.

Elona’s husband had been killed during the Kosovo War for independence from Serbia in 1999. Trying to prevent the small Province of around two million people from seceding, Serbia had declared war against Kosovo in 1998, resulting in the killings of over 10,000 people, with the majority of victims being Kosovar Albanians killed by Serbian forces (Independent International Commission on Kosovo 2000). In addition, 90% of the population was uprooted with 863,000 civilians forced into refuge outside Kosovo and 590,000 internally displaced (Bozo 2001). Throughout the war, torture, rape, and other forms of sexual violence were employed as strategic instruments of terror. In Elona’s home village, Serbian forces committed a large-scale massacre between 24 and 26 March 1999 (International Crisis Group 2000). This was when Elona’s husband perished together with another 239 men, women, and children. Their bodies were removed by Serbian forces from the crime site and dumped into mass graves that continue to be found all over Kosovo and Serbia.

Elona remembered the moment of the confirmation of her husband’s death as the onset of her pains. Similarly, other women I interviewed could pinpoint exactly when their emotional and physical pains had initially started. They explained to me that their symptoms and pain increased when they remembered the war or specific events related to it, or talked intensely about the gruesome events, or visited places where they or others had undergone harrowing experiences. However, they also told me that they experienced the same symptoms when under a lot of stress due to their precarious economic situation, unemployment, inadequate health and social care, and interpersonal conflicts. Indeed, life in Kosovo was marred by insecurity and hardship for many. Since its Independence (17 February 2008), the country has had the highest poverty and unemployment rates in Europe.^2^ These grievances left many people disappointed in the national project with protest erupting all over the country. Such protests were mostly led by men while women’s voices were largely absent from the public discourse. Yet, women did not simply stand by as their country struggled with its history and socio-economic problems. On the contrary, my research among village women shows how they developed and used a shared embodied language consisting of physical and emotional pains to complain, protest, and impact on their environment.

This embodied language has gone unexplored. Rather, women’s painful symptoms have been framed in medical terms whereby traumatic experiences were linked to high rates of psychiatric symptomatology and mental disorders including posttraumatic stress disorder (PTSD), depression, and anxiety (Lopes Cardozo et al. 2003; Morina and Ford 2008). I set out to provide more nuanced insights into women’s illness experiences by exploring emotional and physical pains as a form of communication which I call *SymptomSpeak*. Symptoms, I understand to be more than bodily or mental phenomena, circumstances, or conditions arising from or accompanying diseases or affections (as the Oxford English Dictionary might suggest). Rather, they bring together physical and emotional pains with social and political ills. By “Speak,” I mean an action of conveying information, views, and feelings in interaction with others. It is through such verbal, performative, and embodied interactions that stories can be heard and experienced by others. SymptomSpeak, in turn, lets pain speak as those engaged in such communication search for shared points of embodied and social reference that can render life’s challenging experiences commensurate (the theoretical framework is further developed in the literature review below).

To explore SymptomSpeak among Kosovar Albanian women, I conducted ethnographic fieldwork in two villages, Krusha e Madhe and Pastasel, located in the south-western part of Kosovo over a period of 16 months between 2007 and 2009 followed by annual visits ever since. My research was based on participant observation which allowed me to immerse myself in community life and the daily activities and, thereby, collect data spanning and transcending intellectual, emotional, and embodied realms. These observations I complemented with informal and semi-structured interviews with war widows (24) and non-widowed women (47) of various ages (between 20 and 70 years old) as well as with persons whom they interacted with on a regular basis including health practitioners, traditional healers, religious leaders, members of various humanitarian NGOs, human rights activists, and other key informants such as male survivors of the massacres and eye witnesses, village elders, teachers, politicians, and military personnel.

Illness narratives were elicited informally during conversations as well as more formally through an adapted version of the McGill Illness Narrative Interview (MINI) (Groleau et al. 2006), a theoretically driven, semi-structured interview protocol focusing on basic temporal narratives of symptom and illness experience; salient prototypes; and explanatory frameworks related to labels, causal attributions, expectations for treatment, course, and outcome. Furthermore, the supplementary sections of the topic guide helped me to explore help-seeking and pathways to care, treatment experience, adherence and impact of illness on identity, self-perception, and relationships with others. Semi-structured interviews were audio recorded with consent by the participants while informal conversations and observations were recorded as detailed field notes.

In this article, I focus on the perspectives of women to provide insight into the following questions: What are the linguistic dimensions of pain, and what kind of articulations arise from these painful experiences and expressions? How does the language of pain circulate, connect and reach across histories, gendered realities, and social politics? In what ways might the language of pain act on and transform the world by shaping and changing political and social agendas? I begin by reviewing the academic literature with a focus on the language of pain and, based on this, create my own conceptual framework of SymptomSpeak. Building on this, I present an ethnographic exploration of SymptomSpeak to discuss its potential for new ways of listening, hearing, and responding to pain in contexts of war and hardship.

## The Language of Pain

### When There Are no Words

Pain is usually understood as an effect of, or response to, something—a collision, a physical wound, and psychological trauma (Bourke 2014). Besides a matter of ill health, pain has been explored in terms of its communicative power (Das 2007). Much attention has been paid to the challenges involved in relaying painful experiences to others. Scholars have cast pain as invisible, defying representation and sensory confirmation through others. Scarry (1987) explained that, while pain can be effortlessly known and understood by the sufferer, “even with the most heroic effort it cannot not be grasped” (4). It remains elusive to others, no matter how empathetic they may be. She relates this elusiveness to the failure of language to convey actual experiences of pain as the latter defies, or even “shatters” language. Similarly, others have noted that, in the face of pain, language runs dry (Woolf 1993) or crumbles (Morris 1993).

Pain that results from violence and torture has been specifically cited as an example of incommunicability. For instance, Veena Das (1998) compared women’s experiences of domestic violence with sexual violence experienced during the Partition in India. She argued that women were able to speak about the violence they were submitted to by their husbands more easily—“It was something sayable in [their] life” (181)—whereas the other torturous violence and related pain could not be put into words. The reason was that domestic violence had an everydayness to it for which women had developed a vocabulary and mutual understanding over time, while war-time sexual violence was entirely different in character: it defiled the bodies of women in order to weaken resistance of aggression among men and was, thus, perceived as incapacitating, isolating, shameful, and often unshareable. Pain induced through such horrendous violence, Joanna Bourke (2014) explains, is seen to lack a referential content (unlike other sensations like love or anger that are for or of something) and, therefore, a bridge into the social world where it could be known by others.

### Speaking of Pain

While our language appears to fall short when attempting to communicate pain, this does not mean that there is a shortage of attempts to make pain understandable. In fact, Scarry (1987) herself insists that pain is forced “into avenues of objectification” (6) through retrospective self-reflection by the sufferers as well as in other various contexts such as medical and human rights discourses, art, and the courtroom. One such form of objectivation is the use of figurative language that employs association, comparison, or resemblance to convey experiences resisting expression through “ordinary language” (Bourke 2014). “My stomach is in knots” or “My back ache feels like having an oily rag tied around my spine” are figures of speech used by pain sufferers to explain to others the nature and extent of their pain and, thereby, to transmit interior sensations into the external world.

Terms to characterize pain have also been employed in a more standardized manner so as to describe the subjective intensity of pain experience and render it diagnosable. A prominent example is the McGill Pain Questionnaire first developed by Melzack and Torgerson (1971). Their goal was to refine the pain vocabulary of medicine by gathering the words spoken by patients when characterizing pain. These words were then grouped into classes such as temporal, spatial, thermal, and punctuated pressure and characterized by descriptors arranged according to intensity in order to measure a person’s level of pain over time. Many clinicians and patients continue to consider the questionnaire useful, as it seems to simplify communication between them. At the same time, it was found that the usefulness of the pain questionnaire was not as universal as expected; rather it was highlighted that such pain measures leave out “life’s quirky aspects, its subtleties and nuances” (Dworkin 2014, p. 79), and neglect the fact that being-in-pain does not follow an ideal blueprint within and between cultures. To the contrary, painful experiences are believed to be multifaceted in bringing together attitudes, motivations, belief and language systems, and cognition (Ahmed 2002).

### Acknowledging Pain

Social pain processes have been described as interweaving the personal with the collective explaining that those involved communicate and transmit their pain verbally or through lamentation and bodily expressions as a means to comment on history and present hardship. Ahmed (2002) noted that pain attaches us to one another as it “‘surfaces’ in relation to others, who bear witness to pain, and authenticate its existence” (25). Similarly, Das (2007) states that pain makes claims on the other as it “cries out” for acknowledgement and response (40). Transmitting and sharing affective states between people has a collective dimension to it as people turn to each other for cues and behavioral tools to reproduce and reinforce social relations (Ahmed 2002; Das 2007; Rubenstein et al. 2018). The emerging communities of pain thrive from reciprocity in which pains are exchanged accompanied by multiple acoustic, linguistic, and visual effects (Seremetakis 1991).

Is it possible, however, to feel the pain of others in order to truly authenticate it? What does it take for pain to connect and reach beyond the sufferer to communicate and shape social realities? Grappling with these questions, Das (2007) refers to Ludwig Wittgenstein’s (1958) philosophical thought experiment about whether it is possible for one person to have pain in another person’s body. He writes: “In order to see that it is conceivable that one person should have pain in another person’s body, one must examine what sorts of facts we call critical for a pain being in a certain place” (49). He goes on to exemplify the notion of localizing another’s pain in one’s own body, contemplating:

Suppose that I feel a pain which on the evidence of the pain alone, e.g. with closed eyes, I should call a pain in my left hand. Someone asks me to touch the painful spot with my right hand. I do so and looking around perceive that I am touching my neighbor’s hand (…). This would be pain felt in another’s body. (49)

Wittgenstein invites us to imagine that another person is in pain by perceiving a shadow of their pain on our own body in the locality corresponding to that in which we believe the other is in pain. If one is willing to go along with this thought and *acknowledge* another person’s pain on one’s own body, one has to also somehow *know* that the painful sensation is actually there. Acknowledgement, in turn, requires us to show that one has actually *heard* the message. In other words, Stanley Cavell (2015) writes, as he engages with Wittgenstein’s work: “It is not enough that I know (am certain) that you suffer—I must do or reveal something. In a word, I must *acknowledge* it, otherwise I do not know what ‘(your or his) being in pain’ means” (243). Revealing something can take various forms, among them language and painful embodied reactions that is, an actual “exhibiting of the object of knowledge” (238).

If we accept that pain can transcend bodies and engender communication through bodily and verbal reactions, how might it be possible for those involved to actually grasp the meanings that are communicated through pain? According to Cavell (2015), it would require getting directly to the sensation by moving beyond the outward expression of pain. Yet, he remains skeptical about the actual realization of the endeavor asking how one can so much as try to do this. I decided to take on his challenge by exploring pain as a language capable of transforming symptoms into tangible substances that can be shared through the mediation of relational structures.

### SymptomSpeak: Communicating Through Pain

Painful symptoms can be, among other things, communicative devices that are shared between people in order to convey critical messages about social realities. Borrowing from the philosophers Deleuze et al. (1988), it could be said that if “deteritorialized” from the field of illness, symptoms have the potential to be transformed into utterances as they “reterritorialize” into discursive fields. Reterritorialization, the philosophers explain, is a process in which “deterritorialized elements [symptoms] recombine and enter into new relationships in the construction of a new assemblage [language]” (199). It is, thus, important to pay attention to the movement of components into complex and constantly shifting assemblages. In Deleuze and Parnet’s (1977) words: “We ask each time into which assemblages these components enter, not to which they correspond, nor to which memories or fixations they owe their importance, nor to which incidents they refer, but with which extrinsic elements they combine to create a desire” (97).

What I find compelling about their theory is that assemblages cannot simply be reduced to interconnected structural elements such as particular memories, events, or social relations as would be the case in the biomedical logic where symptoms are attributed to specific causes in order to derive a matching diagnosis and treatment. Unlike diagnostic categories, assemblages do not exist as such, they are not “artefacts,” but have to be captured in the making as they give shape to changing articulations of societal critique and desire. In order to be able to *hear* what symptoms have to tell us, we need to move beyond their multiple causalities and explore the changing relationships and interpretations of the symptoms themselves.

I call such symptomatic communication SymptomSpeak and am interested in what has to happen in order for it to occur. I will show that SymptomSpeak requires dialogic interaction so that utterances do not stand alone but rather in relation to one another. To achieve this, it is important that people involved in SymptomSpeak perceive one another as sufficiently similar in order to be compelled enough to intensify their pain and impress it upon others in the hope of receiving not only acknowledgement, but a response in the same register. The political scientist, Barbara Prainsack, suggested to me in a personal conversation to think of this exchange as an act of “conspirare.” Conspirare means “breathing together” and is also the foundation of the word to “conspire,” that is, the act of collusion between people who secretly plan to do something against someone else’s wishes. I argue here that, through bodily symptoms, SymptomSpeak allows people who perceive each other as similar enough to co-articulate truth claims. This conspiracy of co-articulation serves to question, complicate, and destabilize a status quo that consists of dominant versions of history, social structures, and power dynamics. If acknowledged by others, such newly emerging historical self-understanding has the capacity to shape and change history in that it persistently “sediments” into “macroprocesses” of history-making (Comaroff and Comaroff 1992, p. 38).

Conspiring in SymptomSpeak does not come easy. My research shows that it is hard physical and emotional labor. It is exhausting, sometimes unbearable as the language of pain intensifies and, thereby, materializes not only in the speaker’s body, but in the bodies of the listeners as well. The impressions that these painful dialogs make are not invisible. They leave traces on bodies, minds, and social relations. That is, the sharing of pain ceases to be something aethereal, but instead engages with artifacts of tears, headaches, and cramps that, in turn, historicize exchanges of feelings as they leave marks on bodies and, in this very process, change their meaning (inspired by Seremetakis 1991). At the same time, it would be wrong to portray SymptomSpeak as a heroic act—it is often unsuccessful as it is too painful to endure. It requires pain relief and, in this process, symptoms are subdued and once more “deterritorialised” from the discursive field and “reterritorialized,” albeit changed, into the medical field requiring treatment.

In the following, I will explore these dynamics of SymptomSpeak among Kosovar Albanian women in order to show what kinds of worlds arise from such communication; how SymptomSpeak connects and reaches across histories, gendered realities, and social politics; and how SymptomSpeak changes not only meanings, but also people, social relations, events, and objects in the process of embodied conspiracy.

## SymptomSpeak Among Village Women in Kosovo

### A Symptom Lexicon and the Worlds it Gives Rise to

Initially, I was unfamiliar with the way in which women communicated through their pains and had difficulty letting such embodied communication emerge during our conversations. I asked women questions such as “What kind of health problems do you suffer from since the war?” and received rather vague and somewhat unsatisfactory answers in return. Realizing that I was going about it the wrong way, I began to observe and listen carefully trying to capture SymptomSpeak whenever and wherever it surfaced among the women. Over time, I learned that words such as “stress,” “worry,” or “nervousness” were commonly used to underline ill health, war experiences, the economic situation, or conflicts with family members and friends. To try out my new language skills, I added questions to my repertoire such as “When you get stressed out or nervous, what do you feel?” I then compared the answers that were generated through the different speaks to ensure that I was indeed learning the proper language. The subsequent vignette is illustrative of such language experiments.

Lorida, a widow living in Krusha e Madhe, explained that her most salient problem was her dire economic situation and the fact that she could not always provide her three children with what they desired. Nevertheless, she placed her remaining hope for a better future in her offspring exclaiming, “*Inshala*, [they] will grow up and work one day, and will be able to live on their own without having to beg. It is very difficult to hold your hand open all the time. It is very difficult to always tell your brother or sister, ‘skomje, skomje’ [I don't have, I don't have].” During our interview, she talked about her health in two registers referring to physical health problems and their successful cure and to ongoing emotional distress and pains connected to wider environmental contexts.

*Author: From what kind of health problems are you suffering since the war?*

Lorida: I have had health problems since the war. First, they operated on my gallbladder and then on my eye.

*Author: When you get stressed out or nervous, what do you feel?*

Lorida: As soon as I become nervous, my chest starts hurting. Also, the vertebras in my neck hurt. My doctor told me to go to some hot springs, but I don’t have money.

*Author: What else happens when you get stressed out or nervous?*

Lorida: I get very high blood pressure, very high! Then, I have to go to the doctor to get an injection. My head gets tense and I can’t turn it.

Similarly, other women I talked to listed and displayed an eclectic mix of symptoms during conversations. The following is a list of symptoms, ordered according to frequency in use by widowed and not widowed women in Krusha and Pastasel: Headaches, worries, sadness, blood pressure, nervous, pain, sleeping problems, stress, backpain, eyesight, heart problems, stomach pain, paralysis, neck pain, breathing problems, chest pain, feeling cold, dizziness, eating problems, losing one’s mind, fear, heat, losing consciousness, lack of energy, nightmares, noises in the head, suicidal thoughts, tiredness, claustrophobia, collapsing, cramps, losing one’s head. The list is obviously fragmented as it was teased out of conversations.

Importantly, however, not all of the symptoms were shared or experienced equally between the women with war widows experiencing more symptoms than non-widowed women. Put differently, not everyone mastered or needed to master the same range of symptomatic vocabulary to make themselves heard. When heard as part of narratives, performances, and social interactions, I learned that the differences in symptomatic expressions were shaped by intersecting memories of the past, social experiences, opportunities in life, and passions for social change. During SymptomSpeak*,* bodily pains, emotions, social status, and desires came together and were visualized through bodily expressions, and it was in such moments that topics of discussions were painfully created in concrete social fields and at particular moments in time to sustain memories of the past and reflect critically on present conditions. In the following, I will show how SymptomSpeak allowed women to make their inner states visible and to seek acknowledgement and recognition for their war experiences as well as insecure living conditions and the social marginalization they experienced in their day-to-day lives.

### Evoking SymptomSpeak to Communicate Uncomfortable Truths

#### When You Have Such a Lot of Pressure, How Are You Supposed to Feel Good?

All women I talked stated that they first and foremost experienced pain due to their precarious economic situation. In conversations with me and others, they made their lamentations visible through signs of the body including postures, gestures, and general facial expressions. For instance, I noticed that they would talk about feeling “economic pressure” in the abstract as a sense of “emotional pressure” or a more physical sensation of “high blood pressure.” Abnore sighed, “I always feel pressure” and then asked rhetorically, “When you have such a lot of pressure, how are you supposed to feel good?” Adelina, on the other hand, complained that she had suffered from high blood pressure for nine years and related this to feeling pressure “on her head” to educate her children, and to buy clothes, carpets, and household items. She concluded, “My head is full of pressure.”

Widows often told me that because their husbands died during the war, the ecology of their household had been greatly disturbed and that their means of generating future income had diminished noticeably. Most widows received a widows’ pension of 62 Euros at the time (it was later increased). In order to collect their pension, they gathered at the village bus station each month to drive to the Bank in Rahovec. Dressed in black skirts and dark blouses indicating their mourning, they waited in front of the bank complaining about being humiliated by only receiving 62 Euros. Once they received their money, they left to buy items for their household. By the end of the afternoon, most of their pension had been spent on household items. Once at home, many women developed headaches and complained about high blood pressure, felt unable to work in their houses and fields, and could hardly sleep during the night. On one such day, I visited Makfire who looked tired, pale, and worried. When I asked her what the matter was, she said: “One can only get sick going there to get 62 Euros. What do you do with 62 Euros when you have children to feed? Nothing! It’s not fair that old people get a pension of 42 Euros and we, who have to raise children, get only 62 Euros. (…) When you get the money, you buy a few things for the household and a bit of food. After that, hardly anything is left and the next month hasn’t even started.” She repeated, “We all get sick when we have to go there.”

Similarly, other women related their physical and emotional pains to economic problems and, connected to this, the inability to raise their children in a secure environment, widowhood, and humiliation. These ever-present situations were experienced as grueling and demoralizing. Sometimes, they were just little inconveniences or outwardly manageable obstacles; yet these “little things” piled up to almost insurmountable difficulties as they chipped away, little by little, motivation, inner strength, and health.

#### We Are All Destroyed Since the War

When asked when they had experienced their symptoms for the first time, women tracked the onset of their currently experienced symptoms to the war. Several women attributed the onset of their symptoms to the harrowing experiences of being separated from the men before they were shot dead by Serbian forces. Other women referred to their flight and uncertainty of whether their family members were alive or dead. Again, others associated the onset of their symptoms with conflicts that arose in their families due to the crowded conditions in the refugee camps or attributed them to the shock they experienced when seeing their destroyed villages on their return from refuge.

Sharing stories about the onset of symptoms was not only an illness narrative, but an opportunity to provide testimony. To understand the symptoms’ role in establishing historical truths further, I probed by asking whether they had suffered from similar symptoms before the war when they got stressed out or nervous. In their responses, almost all of them clearly distinguished between a healthy life before the war and a life characterized by sickness, economic difficulty, and interpersonal conflict since the war. They drew a clear line between *para luftës* (before the war) and *pas luftës* (after the war) and, thereby, gave the origins of symptoms a space and time.

Particularly for widows, life had radically changed after the war. Widowhood was considered a watershed moment and related to ill health. The women equated their husbands with a life in which they wore beautiful clothes and abundant jewelry, used makeup, had plenty of money, and, above all, were completely healthy. Adelina said passionately, “I was never nervous. I was always dressed up, wore makeup and I didn't care about the rest. Why should I have been nervous? I had my husband, my children, our two houses, I had clothes and food. But now after the war, I have only worries.” Everyday life since the war was often experienced as unbearable by widows not only due to war related memories, but also because of communal expectations which prevented them to go out for pleasure, dance during celebrations, wear colorful clothes, and be publicly joyful. Some women referred to such customs as “stupid Albanian traditions,” “uncivilized,” or “oppressive.” Lorida angrily flung her hands into the air and shouted when talking to me about these constraints, “As widows we can’t do anything! At weddings we can’t dance. We are not allowed to be happy! Alright, our husbands died, but, do we have to die as well? No!”

While customs and societal rules were experienced as oppressive and distressing, it is important to note that the women were efficient at bending or ignoring them. Moreover, the rules were not timeless. When I returned ten years after the war, many widows took the anniversary as an opportunity to shed some of their dark clothes and introduce color into their wardrobes, dance with their children at festivities, and explore cafes and restaurants with their friends in nearby towns. History is, after all, constantly remade through the body and concrete social interactions and points in time.

#### Serbs Not Only Killed Our Husbands, They Also Killed the Friendship Between Us Women

Symptoms were not only used to express war experiences and economic hardship—they also told of social conflict between women and their communities over limited resources and social morality. Symptomatic narratives circulated to foster competition, express blame, cast in-group criticism, and evaluate and re-evaluate behavior and personalities through gossip. To illustrate the strength of SymptomSpeak, let me refer to an example featuring a widows’ collaborative that worked to process and sell peppers.

The group of widows had received support from an international non-governmental organization to set up their business over several months. Everything appeared to be running smoothly until group members discovered the corrupt practices of one of their leaders. The women felt betrayed and angry, developed headaches and stomach pains, and as a result lost the motivation to continue working. When Teuta could not handle her pains anymore, she decided to visit the private clinic of a doctor where she received injections “to calm down.” Yet, despite the medication, her health did not improve. In conversations with me and other members of the group, she related her emotions and physical reactions to the conflict in the group while also building links to previously experienced pains when the mortal remains of her husband were discovered and brought back to her after the war. She told us, “I know the feeling of stomach pain and nausea from before. I had the same symptoms when the remains of my husband came back. Then, I felt awful, could not eat, had stomach pain and felt nauseated. I know that I get these symptoms when I am stressed and worried.” I asked what the doctor had advised her to do and she responded,

Well, when I went to him, he just said that I should stop being so sad, accept that I lost my husband and find ways to go on with my life. He thought that I had come for *trauma*. But, of course, I couldn’t tell him that I was suffering from the problems in our group. I could hardly have told him that it’s because of [name of co-worker] and that I got nervous because she stole from us. Imagine, he would tell his wife and then…

It was not the first time that women had explained to me that doctors treated them for *trauma* or, as they often phrased it, *post-trauma* when they were unwilling to explain the source of their problems for fear of the spread of gossip and rumors in the community. Beyond gossip, the example also speaks to the lack of solidarity not only among the women, but also the community. Widows especially often complained about a communal hypocrisy in which they were put on a pedestal and valorized as the wives of martyrs while at the same time receiving little in terms of support when dealing with acute economic difficulties and working conditions. Within this context, feelings of helplessness and loneliness could be overpowering leading to worries, headaches, stomach pains, and nausea.

## Conspiring in SymptomSpeak to Shape and Change Social Realities

While I was often concerned about the women’s health and wellbeing, I also learned to appreciate their embodied articulations as having a dialogic function that brought women together to conspire to shape themselves and their communities despite seemingly insurmountable social, economic, and political barriers. In the following, I will continue to explore the shape that SymptomSpeak takes through evolving dynamic assemblages, labeled as *nervoz* (nervousness), *mërzitna* (worried, sad), *mzysh* (evil eye) and *t’bone* (spell). I will present the assemblages as “cases” along worldly and magical lines in order to describe how women variously combined components of SymptomSpeak and, thereby, created multiple discursive patterns that manifested through a combination of embodied and verbalized expressions in particular social contexts.

### The Politics of Nerves

“Nerves” was a prominent discursive pattern among women that was used to discuss and challenge interpretations of the past and current economic difficulties and power hierarchies. In colloquial Albanian, *nervoz* has two meanings. The first refers to a sentiment of irritability and anger. One can be *nervoz* about someone or something without developing symptoms that affect one’s health. However, *nervoz* had a second meaning pertaining to bodily and emotional pains. When I asked women whether they had a certain term or expression that encapsulated their symptoms and related experiences, each one of them replied that they suffered from *nervoz*. Adelina explained, for instance, “Lately I develop high blood pressure when I send my son to get the money for the installments for the credit [a micro-credit project in the village]. I become *nervoz* and develop high blood pressure. So, I know it's from *nervoz* as my heart starts trembling and beating strongly.” Adelina attributed *nervoz* both to social events—the concern of not having enough money left for the rest of the month after paying off the installment for credit as well as bodily conditions including high blood pressure, trembling, and a strongly beating heart.

Women often said that they only become *nervoz* about matters that are important to them. These included memories of the war, related grief, the burden of widowhood and the overwhelming responsibilities associated with it, economic problems, the fear of not being able to provide for their children, and anger due to interpersonal conflicts. For instance, S. Sh^3^ explained during an interview, “When I work hard, I don’t experience *nervoz*. But when I think about the war, I do. But lately it happens also when I don’t think about the war. When I get *nervoz* with my children, it appears as well.” This was also true for others who explained that symptoms increased when they remembered the war or specific events related to it and visited places where they or others had encountered gruesome experiences.

*Nervoz* was considered to be a wavering entity that rose and fell in connection with the combined effects of individual and collective memories, social context, and emotions. Like the tide, it could vary in intensity and timescale, ranging from hours to years as it was subjected to various forces. After her husband had returned from Germany, where he had lived and worked for one year to contribute to the family income, Linora explained, “I am freer and my *nervoz* is less. Now that my husband is back, I feel much better.” However, since her *nervoz* was connected to whether her husband was present or not, she continued, “But if he goes again my health will change and the problems will be back.” Bodily reactions thus mirrored the fluctuation of everyday life, which was often perceived as out of one’s control. Talk about *nervoz* was one way to articulate these insecurities and complain about inequalities and the status quo.

### Spinning Sadness and Worries into Moral Webs

Another assemblage of bodily, emotional, and social pains was labeled *mërzitna*. *Mërzitna* is the popular version of the word *mërzitem* and is translated as to be sad, worried, or bored. As such, it is not an illness category but an ordinary word pointing to feelings of uneasiness, nagging concern, or listlessness. However, if a situation or feeling becomes unbearable, *mërzitna* turns into an expression of distress.

Women evoked *mërzitna* to convey their sadness in relation to loss and loneliness in the context of war, the death of a family member or friend, or husbands and children moving away from home to work abroad or to study in Prishtina. When I arrived at Teuta’s compound, she looked tired and worn out. Leaning against my car she said that she could hardly sleep last night and that her entire body was hurting. She sighed, “I am *mërzitna* that’s why every bone in my body hurts.” When I asked her why she felt *mërzitna*, Teuta explained that she had given an interview to Polish humanitarian aid workers the day before that had reminded her of past sadness and hardships. “The Polish women are in Kosovo for only four days and try to talk to as many women as possible. The interview lasted for two hours and was all about the war and the way I coped without my husband. I wasn’t prepared for it and, before I knew it, war memories awoke inside me.”

*Mërzitna* was not only evoked by concrete events, but also during times of idleness, when hands rested and distressing thoughts intruded keeping the women in their grip. Especially during the wintertime, when there was little farm work, women had recurring thoughts that circled in their minds blending memories of the war with current worries about their economic situation. One woman said, “If I sit and drink coffee all day long, I start to think about the sad things, about the war, about the people we have lost. I become *mërzitna.*” Another explained, “[in the summer] I work in the garden and around the house; there is lots to do. But now, I drink tea, coffee, tea, coffee… When I get lonely, I cry.” Feeling lonely and heartbroken was related to the pervasive character of sadness, grief, and worries.

Discourses on the notion of “feeling worried” differed from those related to sadness. Feeling worried went along with a sense of compassion in that the women felt sick worrying for somebody else’s wellbeing. In so doing, they accentuated a positive image of themselves by talking about their bodily and emotional symptoms within the context of their deeper concern for family members. For example, Arieta felt weak and nauseated worrying for her son who had injured himself on his construction job in Italy and Flora suffered from undefined pain as her daughter was going through a complicated pregnancy. Such SymptomSpeak was composed of worries for others, rhetoric self-sacrifice and bodily pains, and helped to form moral webs between individuals, communities, and institutions. These webs, in turn, can be considered to function as fragile safety networks into which individuals and groups could position themselves relatively safely when casting moral judgment on their country’s dire economic situation, political mismanagement and family disintegration due to labor migration.

While such SymptomSpeak often appeared somehow selfless in that women demonstrated how emotionally invested they were in the lives of family members and friends, it was also important that this act did not go unnoticed by others. Sharing their stories ensnared individuals and communities in such a way as to guarantee exchange of “shared substances” including symptoms, emotions, and material goods. Individuals taking advantage of this moral network without feeding into it were, in turn, judged as greedy and inconsiderate, often leaving the other party feeling disappointed. This became particularly clear when an older lady complained bitterly to me that she had sacrificed herself for her family-in-law before and during the war and received hardly any recognition for all she had done. She said: “They were like ‘now that we crossed the river with the help of the horse [her]—screw the horse as we don’t need it anymore’.” The example highlights that the dramas of the everyday could be overwhelming and the moral webs insufficient in providing support and a sense of security. Overcome by feelings of sadness and worry, women brought to the fore dissonances between self and society.

### Creating Circles of Exclusion and Social Control Through the Power of the Eye and Spells

Just like *nervoz* and *mërzitna*, I consider *msysh* (evil eye) and *t’bone* (spell) assemblages of SymptomSpeak that are dynamic and ambiguous and open to interpretation and negotiation within its sociocultural context. *Msysh* generally refers to the “voluntary” or “involuntary” ability of the human eye to cause harm when directed at other individuals and their valuable possessions in admiration, envy or jealousy. Those affected often experienced feeling *mërzitna*, strong headaches, nausea and vomiting, weakness, problems with breast feeding and the sensation of heat moving up their bodies.

People suspected that those who cast *msysh* on others were people with heightened desire. Although anyone could involuntarily send out *msysh* when admiring someone greatly or being envious or jealous, I found that, in many cases, maternal family members were suspected of such deeds. That is, evil eye brought family relations to light that tended to threaten the patriarchal order of things by giving more influence to maternal kin (i.e., through frequent visits, close friendship, or financial dependency) than the formal kinship system foresees. Indeed, the formal system devalues maternal relations in order to further strengthen the paternal ones as is illustrated by the fact that the male or paternal line is referred to as a blood or thick line and the female or maternal line as a milk or weak line (Kaser 1995). The fear that maternal relatives would hold more influence than they were entitled to seem to be connected, not only to familial relations, but also economic insecurities. Research conducted in the region highlights the importance of smoothly functioning family networks when state support is absent or hard to reach in order to ensure “better and quicker access to resources” as well as “reliable protection against elusive and threatening conditions in a rapidly changing socio-economic environment” (Schäuble 2014, p. 273).

While *msysh* was a mode of expressing distress over unhinged social relations, it also served as a means to deflect from the women’s own responsibilities, choices, and personal difficulties, by projecting their children’s health problems or their own distress onto external stressors. This is how narratives on *msysh* created alternative meanings and messages, depending on the context, and enmeshed persons further in moral webs exposing them to moral judgments. It was also a delicate balance of keeping certain individuals at bay and justifying social exclusion for the sake of one’s own wellbeing, while, at the same time, exposing oneself to the same possible fate. Negotiating these uncertainties was not straight forward—just like anything else in the everyday life of a post-war and economically unstable society. Consequently, I regard *msysh* as symptomatic of wider socio-political problems that affected not only the women, but also the communities in which they lived.

*T’bone*, on the other hand, is an external force of distress and another form of SymptomSpeak. Unlike *msysh*, it is considered to be black magic that is cast purposefully by a person on another in order to destroy social relations, cause material harm, and lead to psychic distress and physical pain. Spell translates into Albanian as *magji* (magic) and to cast a spell into *bëj magji* (to do magic). In colloquial language a spell is referred to as *t’bone* or simply *bone* and is considered a form of *magji e zezë* (black magic).

Victims suffering from *t’bone* commonly experienced a wide range of symptoms such as losing one’s mind, losing one’s knowledge, not knowing who one is and where one is, not knowing what one is doing, losing consciousness, feeling heat moving up in one’s body, feeling extremely *nervoz,* strong headaches, losing control over thoughts, talking dirty, pulling one’s hair, scratching one’s face, infertility, avoiding family members, failing to prosper in material wealth, and being entangled in a chain of unlucky events such as automobile accidents, episodes of sickness, failing school, long phases of unemployment, not being able to accomplish tasks etc. Culprits casting *t’bone* were believed to be solely women and I was often told: “Only women can do such evil things.” Usually these women were mothers-in-law, sisters-in-law, elderly neighbors, or deceitful friends.

Women who were accused of witchcraft were believed to turn to traditional healers and, sometimes, to *shehs* in order to commission evil amulets, knotted black and white threads or items that had been cursed through song such as an article of clothing from the chosen victim, the water of the dead or from the blacksmith, eggs, lipsticks, crochet needles, mercury, and blood. The cursed items were then hidden in the victim’s house in attics, under couches and pillows, under or above door steps, in door hinges and behind door knobs, sprinkled on clothes, mixed in coffees, spread on the floor of kitchens or bedrooms. When Flora’s sister-in-law started to pull her hair, tried to squeeze out her eyes, and scratch her face during her father-in-law’s farewell celebration before traveling to Mecca, it was found that the water of the dead had been sprinkled on her *demi* [traditional dress]. The water of the dead is water that women collected secretly when a corpse was washed by a *hoxhenica* before the burial ceremony. Such waters were considered highly dangerous, and when used as a curse, I was told that “you will never have a good day in your life.”

Several of the women agreed that cases of *t’bone* had increased since the war. According to them, the reason was due to an increase in gossip and rumors, jealousy, and uncertainty. Gossip and rumors led to suspecting others of misconduct and were a means by which events that were difficult to explain and deal with were pinned on people who one despised or had difficult relations with. Such actions were perceived to be carried out in defense of moral values and “truth” and, thus, became seen as justifiable. Through these means, vicious circles of exclusion and social control were created that were hard to break if one was entangled in them. It is, therefore, important to understand that SymptomSpeak is not merely a way to communicate suffering and injustice; it could also be turned into a device capable of harming others while enhancing one’s own status even if only at the margins. In other words, pain is not simply passive or vicarious. It is related to and impacts on the politics and power of the everyday.

## Discussion

In Kosovo, SymptomSpeak is a painful language through which women “breathed together” that is, conspired in order to articulate, through symptomatic utterances, truth claims that defiantly questioned, complicated, and destabilized dominant versions of reality. In this article I showed how symptoms, as communicative devices, were shared and exchanged between women as they entered into relationships with one another as well as the wider social and political world. The emerging assemblages served not only as commentary on, or representations of the history and present realities but acted on and transformed the world the women lived in. In other words, they had a bearing on dominant power hierarchies, political agendas, and social relations in families and the wider community.

Yet, speaking SymptomSpeak was not at all easy and having one’s message heard in public even harder. It was an ongoing visceral, emotional, and social struggle for women in rural Kosovo. Their views and voices were mostly pushed to the side by the Kosovo State, communities, and families as the public sphere was mostly a space reserved for men to outline grievances and voice their outrage about, and demand justice for, war crimes (Krasniqi 2011). Consequently, women’s narratives were rarely fully acknowledged by others as they were rendered non-representative and denied sought after recognition (Krasniqi 2007). Such gendered narrative asymmetries are of course not particular to Kosovo. Rather, they form a crucial part of societal structures as they are “critical to socializing prevailing ideologies” (Ochs and Capp 1996, p. 33; Povinelli 2011). Prevailing ideologies about the past and present leave little room for questioning, alternative versions, and dismantling nationalist narratives (Humphries and Kalili 2007), and those who dare to perceive things otherwise are brought back into line through various forms of social pressure such as blackmail, shaming, and gossip (Das 2007; Povinelli 2011; Seremetakis 1991).

It is, thus, challenging for those at the margin to find their voice and receptive audiences ready and curious enough to engage with their non-conformist and antagonistic tales. I have demonstrated that women in rural areas of Kosovo bravely took on this challenge. They conspired and breathed together in order to develop, refine, and share SymptomSpeak to formulate their discontent with the past and present. I suggest that such embodied storytelling was a convenient, if not strategic choice in that it protected the women from blackmail and shame as they could camouflage their narratives about socially contentious issues as illness stories. While their illness stories about “very high blood pressure” or “splitting headaches” generated empathy, medical advice, and humanitarian aid among outsiders, it simultaneously allowed the women to communicate critically about history, gendered realities, and social politics as they tried to shape and change social and political agendas on family and community levels.

The characteristics of SymptomSpeak are not easily captured, understood, and spoken by insiders, let alone, outsiders. I, for my part, had to learn painfully that it requires engaging with something that resisted my thinking and feeling. First, SymptomSpeak goes against the impulse to follow a “biomedical logic” by interpreting painful symptoms as signs of illness and to translate the latter into recognizable and treatable illness categories (Giordano 2014). Second, it questions widely held presumptions that pain is a private sensation and that it is accordingly impossible to share. Rather, I learned that for SymptomSpeak to occur, it was important to feel another person’s pain, to have pain in another person’s body, and to unravel the meaning of symptoms by going beyond notions of ill health.

As I was exploring these avenues of feeling, thinking, and hearing, I often felt dragged in opposite directions as the narratives pulled speakers and listeners simultaneously into bodily and socio-political fields. It required the ability to concurrently move outward retracing steps through winding paths from narratives about symptoms, to actual expressions of pain, to attributions of causality while, at the same time, moving towards the center by unraveling sources of pain, discerning uncomfortable truths, and hearing declarations of desire for meaningful futures. At first, the pulling movements confused me as I tried to follow and keep up with the women as they navigated this maze through branching passages and overlapping movements connecting events and processes that affected their livelihood and overall wellbeing. Traversing this maze evoked feelings of fears and anxieties as I listened to echoes of the past, worried about getting hopelessly lost in the present while also feeling uncertain of what I might find at any given crossroads. Yet, mazes are not just about fear and feeling lost—they are just as much about solidarity where people can share, exchange, and listen to each other’s experiences and engage in, not always compatible, meaning making processes (Hautzinger and Scandlyn 2017).

As SymptomSpeak became more accessible to me, I began unpacking the symptomatic vocabulary and its inherent characteristics while trying not to lose sight of its painfulness. Symptoms, in their multiplicity, expressed intimate feelings of loneliness, sadness, loss, and hurt as well as yearning for a past associated with solidarity, wellbeing, and celebrations of life. At the same time, the symptomatic stories ventured outward discussing unsanctioned themes such as dissent with dominating historical and political constructions of reality, the iatrogenic effects of well-meaning humanitarian aid, and competition for scarce resources among persons with equal needs and desires. SymptomSpeak also created space for bold commentary on the burden of widowhood and lack of societal support, the need for husbands to work abroad to support their families despite the promises made for an improved economy in the newly independent State, and unstable and problematic family relations and friendships marred by jealousy and gossip.

Dynamic assemblages like *nervoz*, *mërzitna*, *mzysh*, and *t’bone*, in turn, enabled women to create discursive fields that brought these seemingly disconnected themes together into formations of critique of societal structures, power, and morality and the ways in which these forces impacted on their daily and often precarious lives. In this process, the personal became a collective experience that brought into its fold wider social and political dimensions of public life to be worked on and transformed so as to bring about wider- reaching societal change.

This form of engagement with inner and outer worlds was a painful one and, to some extent, self-destructive as it required arduous physical labor that left traceable scars on the women’s bodies. It slowed women down, bound them to their couches, frustrated interactions with their children, and meant that work was left undone. To endure their sickened bodies and be able to carry on, women often felt the need to reframe their pains into health problems and to request pain killers and “calming pills” to subdue their pains and, in this very process, silence themselves (AUTHOR). Thereby, symptoms re-entered the realms of illness in order to be dealt with through biomedical logics and related forms of treatment. These oscillations between communicative and medical fields weakened SymptomSpeak as it was often misunderstood by medical practitioners and humanitarian aid providers as one-dimensional concepts of trauma, grief, and signs of eligibility for aid. “When I went to [the doctor], he just said that I should stop being so sad, accept that I lost my husband and find ways to go on with my life. He thought that I had come for *trauma*,” Teuta had explained. Due to such limited understandings or misunderstandings, messages conveyed through SymptomSpeak achieved little radical societal changes.

This reminds me of what Kleinman (1994) noted among survivors of China’s Cultural Revolution who employed their symptoms of neurasthenia as rhetoric of complaint about the brutal conditions of the Cultural Revolution and other socio-political sources that they blamed for their misfortune. Yet, despite the clarity of articulation, their symptoms remained “hidden transcripts” that is, ineffective “means for constructing a collective discourse of wretchedness that was critical of the state and that could challenge its policies” (175). In the case of Kosovar village women this also seems to hold true, at least when focusing on macro-politics and economics. Their stories had limited effect beyond the personal sphere in that recognition had to be bestowed from the very social fields that were perceived, at least to some extent, as accountable for having robbed them of their loved ones, dreams, and aspirations for the future. However, this is not to say that women’s narratives had no effect. When focusing on the “micropolitical dimensions of social change” (Patton 2011), I would argue that the women were not completely without influence considering that research has shown that “history involves the sedimentation of micropractices into macroprocesses” (Comaroff and Comaroff 1992, p. 38). For instance, they enabled many (although not all) of their daughters and sons to lead different lives by attaining a higher education, increasingly establishing marital relations based on love rather than having to accept arranged marriages, working (job availability permitting) while still being able to raise children in familial environments, and enjoying life without having to constantly justify themselves. Due to the different realities they managed to live in, the new generation did not need to invoke the same kind of SymptomSpeak as their mothers to articulate frustrations within their social worlds. SymptomSpeak is thus neither universal nor timeless. What may appear to be relatively stable assemblages such as *nervoz*, *mërzitna*, *mzysh,* and *t’bone* are, in fact, dynamic as they build up, collapse, or simply melt away, and shape shift in other contexts.

In conclusion, painful utterances attach us to others not by mere “perspective taking” whereby we imagine the world from someone else’s vantage point or imagine ourselves in someone else’s place, but through affective “cross-over” whereby affects are transmitted from one person to the next (Clark et al. 2019). This understanding comes with responsibility. Levinas (1988) reminds his readers when writing that the experience of pain can only be rendered meaningful in the inter-human that is, the “non-indifference of one to another, in a responsibility of one for another” (165). Conversely, Das (2007) writes that the denial of the other’s pain “is not about failings of the intellect but the failings of the spirit” (57). Therefore, for us to experience the other’s pain, we not only have to provide it a home in language but also in our bodies so as to be able to conspire in finding forms of mutual care which allow for healing through the creation of new historical self-understanding, justice, and meaningful societal change.

## Footnotes

1 All names are anonymized.

2 Statistics from the World Bank indicate that around 45% of the population lived in poverty and around 15% in extreme poverty (UNDP 2012; World Bank 2006, 2009).

3 The woman asked me to use her initials instead of a pseudonym when writing about her experiences.
